# Immediate Implant Placement by Interradicular Bone Drilling before Molar Extraction: Clinical Case Report with One-Year Follow-Up

**DOI:** 10.1155/2018/6412826

**Published:** 2018-04-01

**Authors:** Stuardo Valenzuela, José M. Olivares, Nicolás Weiss, Dafna Benadof

**Affiliations:** ^1^Postgraduate Implant Dentistry Department, School of Dentistry, Universidad Andres Bello, Santiago, Chile; ^2^School of Dentistry, Universidad Andres Bello, Santiago, Chile

## Abstract

The placement of immediate implants in the posterior sector is a widespread procedure where the success and survival rates are similar to those of traditional protocols. It has several anatomical challenges, such as the presence of interradicular bone septa that hinder a correct three-dimensional positioning of the implant and may compromise primary stability and/or cause damage of neighboring structures. The aim of this article is to present the treatment and the one-year clinical follow-up of a patient who received immediate implant placement using an interradicular bone-drilling technique before the molar extraction.

## 1. Introduction

Immediate implant placement has considerable advantages over the conventional approach. It has fewer numbers of surgical procedures, reduces overall treatment time, and therefore costs less. It also helps preserve the gingival architecture and increase the patient's comfort, acceptance, and satisfaction [1–5].

Immediate implant placement studies in the esthetic and premolar zone follow strict surgical protocols that have been established to optimize the three-dimensional positioning of the implant and its primary stability and the condition of the neighboring tissue [6–8]. However, there is less information on immediate implant placement in the posterior sector where the esthetical impact is lower, but the surgical difficulty of the tooth extraction, drilling, and implant placement is greater [9–11].

Despite the abovementioned issue, the cumulative survival rates reported for immediate implants placed in molar sites are similar to those placed in healed sites, which ranges from 93.9% to 99% [4–8, 10, 11]. An essential aspect to achieve this positive outcome is the primary stabilization of the implant in the apical and/or lateral bone, where anatomic conditions can hinder this goal. Therefore, a thorough implant surgery planning, skills, and clinical experience are relevant factors in the success of the surgical procedure [4, 12].

Modifications to the current surgical techniques are recommended to facilitate immediate implant placement in the posterior sector. Different authors propose implant drilling prior to tooth extraction in order to stabilize the interradicular bone septa through the remaining tooth roots [9, 13–15]. In 2017, a randomized pilot study of 22 patients compared the conventional technique of dental extraction, subsequent interradicular bone drilling, and immediate implant placement to the technique of interradicular bone drilling using ultrasound devices. The results were statistically higher for the implant positioning and primary stability using the proposed new technique [9].

The aim of this article is to present the treatment of a patient by means of immediate implant placement using an interradicular bone-drilling technique and its clinical follow-up one-year later.

## 2. Case Presentation

**Figure 1:**
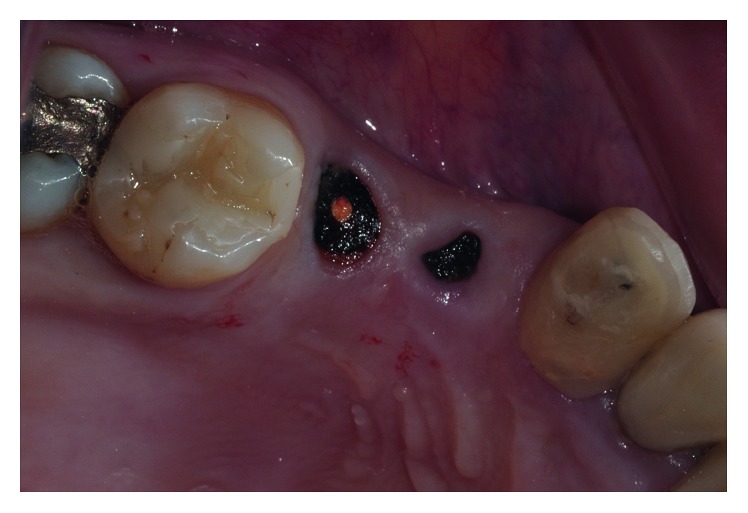
The remaining roots in teeth 1.4 and 1.5 presented decay and exposed endodontic treatment. Tooth 1.6 had an extensive crown restoration and deep, subgingival distal decay.

**Figure 2:**
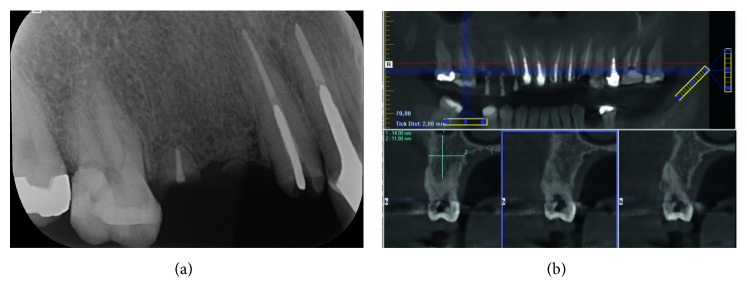
(a) Preoperative radiograph. (b) Upper maxillary CBCT of Tooth 1.6.

A 35-year-old patient with no significant medical history consulted the Implantology Department of Universidad Andres Bello in Santiago, Chile, for a complete evaluation and dental treatment. The dental team performed a clinical examination (Figure 1) and a radiographic study (Figures 2(a) and 2(b)) on the patient detecting decayed remaining roots in teeth 1.4 and 1.5 and performed an extensive restoration presenting deep, subgingival distal decay on tooth 1.6. Based on all the gathered information, the dental team decided to extract teeth 1.6, 1.5, and 1.4 and then perform immediate implant placement.

**Figure 3:**
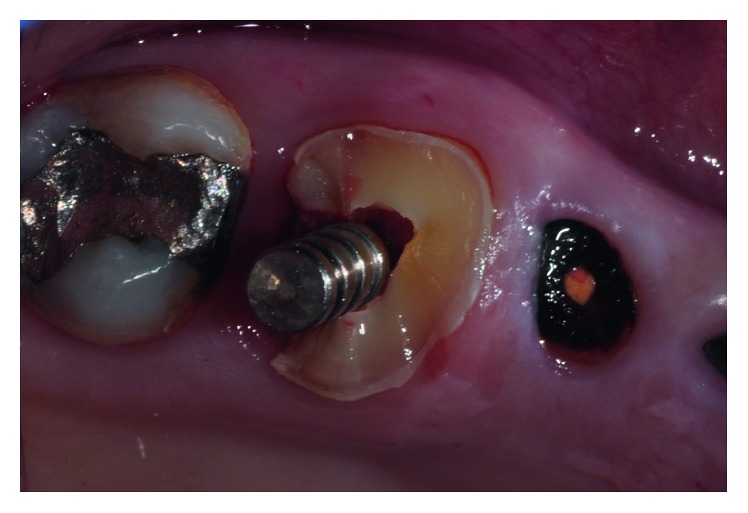
Implant paralleling pin inserted after interradicular bone drilling.

**Figure 4:**
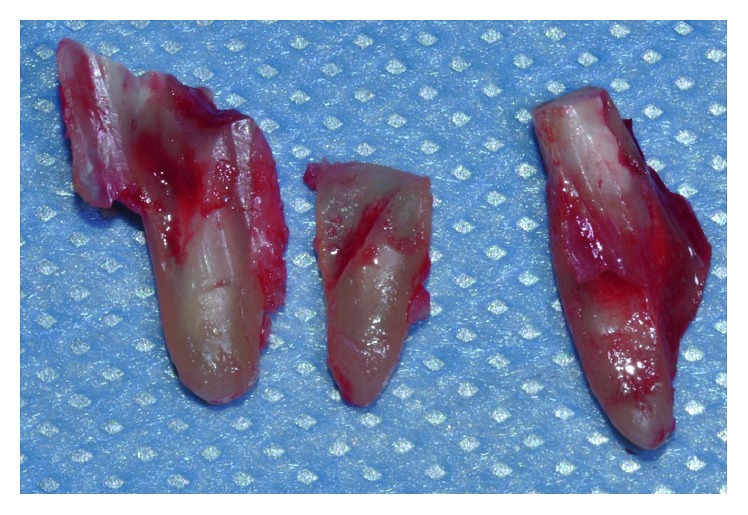
Remaining root aspects were carefully extracted using desmotomes.

**Figure 5:**
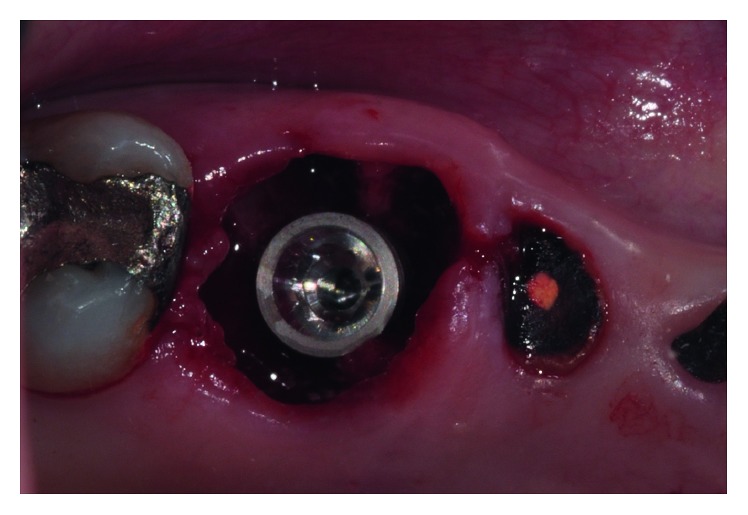
Immediate implant placement of an Alpha Bio ICE 5.3 × 10 mm implant in fresh extraction socket.

Before the surgery, the patient signed the informed consent. The surgical procedure for tooth 1.6 began with the infiltration of local anesthesia (standard Lignospan, Septodont) in the treatment zone. Then, the tooth was decoronated at the gingival margin level using a cylindrical AV-010 diamond burr (Beavers Dental, Kerr Corp). Once the roots were clinically visible, the drilling sequence recommended by the implant manufacturer was performed through the tooth, always corroborating the drilling direction with a paralleling pin (Figure 3). When the drilling sequence was completed, the remaining root fragments were carefully removed using desmotomes (Figure 4). This procedure was done with extreme care in order to preserve the alveolar walls and avoid bone deformation at the drilling path. The alveolus was carefully cleaned and washed surgically with a saline solution, and an Alpha Bio ICE 5.3 × 10 mm implant (Alpha Bio Tec.) was placed in the center of the interradicular bone, in a type B position according to the Tarnow classification (Figure 5). To promote a nonsubmerged healing approach, a standard healing abutment of 4.6 mm of diameter and 4 mm of length (Alpha Bio, Alpha Bio Tec.) was connected to the implant. The 3 mm horizontal gap between the implant and the bone walls was filled with a xenograft (Alpha Bio's Graft, Alpha Bio Tec.) Finally, platelet-rich fibrin (PRF) membranes were fixed through a monofilament nylon blue suture No. 5/0 (Tagum 2/0 HR25, Tagumedica S.A.). Additionally, Alpha Bio ICE 3.7 N × 10 mm implants were placed in teeth 1.4 and 1.5 (Alpha Bio Tec.) (Figure 5).

## 3. Short- and Long-Term Follow-Ups

**Figure 6:**
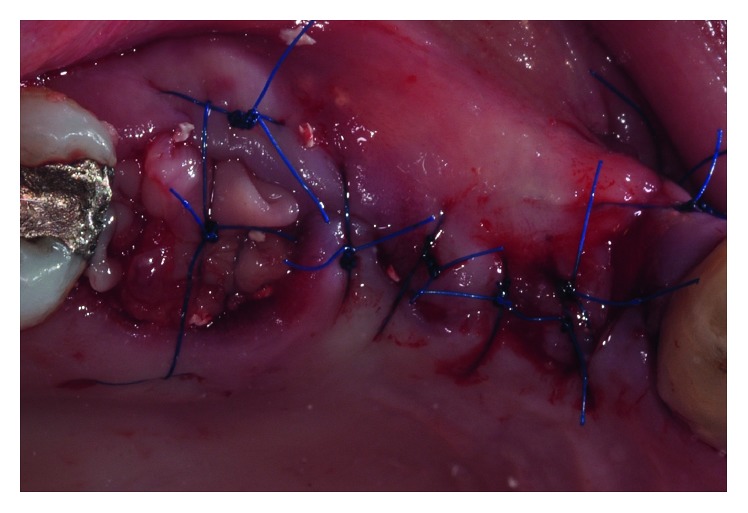
Closure using a 5.0 nylon suture.

**Figure 7:**
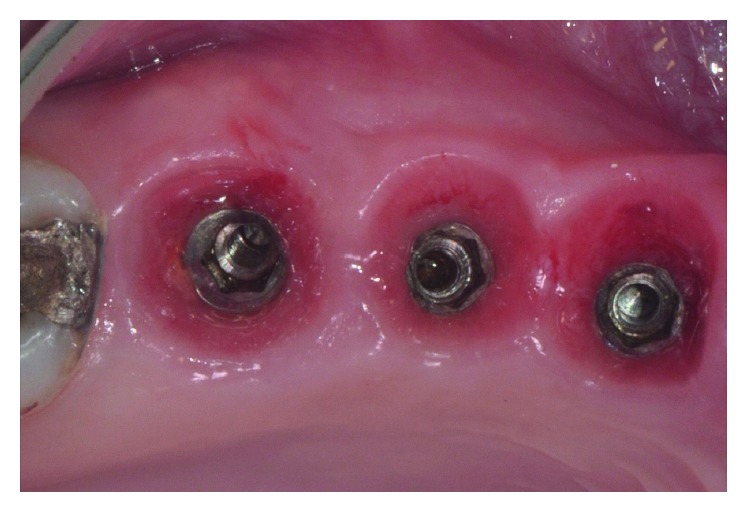
Follow-up at 6 months.

**Figure 8:**
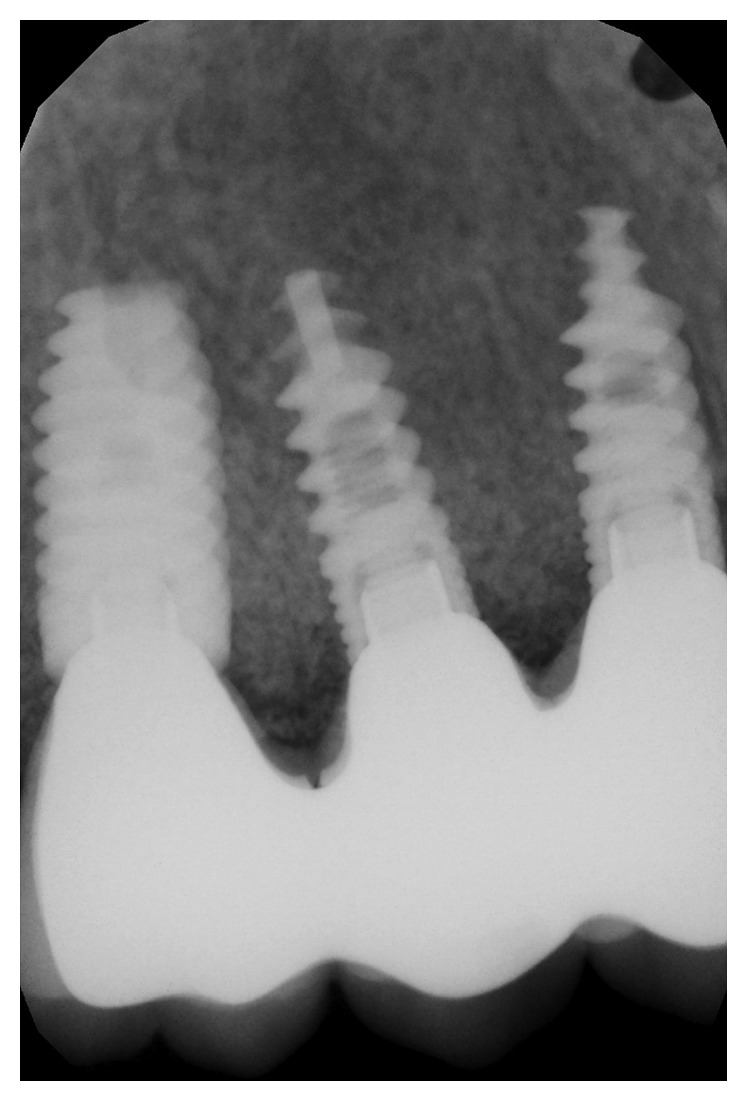
Prosthetic delivery radiograph.

**Figure 9:**
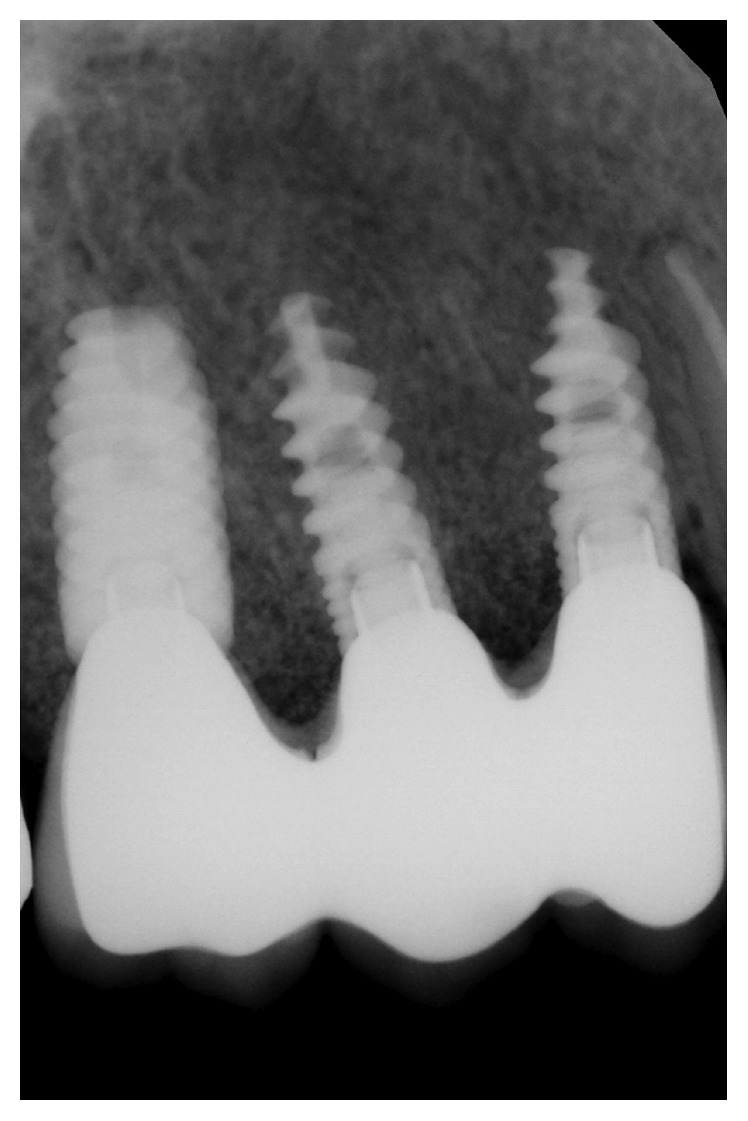
One-year follow-up.

The patient received regular check-ups on days 3, 7, and 14 after the implant placement surgery, and no pain or infection was observed. On day 21, the suture was removed and the soft architecture preservation looked uneventful. At 6 months, an apical reposition flap was performed at implants placed in teeth 1.4 and 1.5, and healing abutments were placed (Alpha Bio, Alpha Bio Tec.). After the soft tissue healed, a rehabilitation based on fixed partial metal-ceramic denture screwed on UCLA Cr-Co abutments was installed. At this time, the gingival architecture remained stable with preservation of a functionally attached gingiva (Figure 6). At 6 months and 12 months follow-ups, clinical and radiological exams showed that the bone levels remained stable (Figures 7 and 8). In addition, the prosthetic structure remained in optimal clinical condition, displaying optimal esthetic and functional results (Figure 9).

## 4. Discussion

The immediate placement of dental implants is a widely accepted procedure, achieving survival rates comparable to implants installed according to conventional treatment protocols [2, 9]. Although there are standardized protocols and numerous studies describing this technique in the esthetic zone, there is less information about the installation of immediate implants in the posterior sector where the esthetical impact is lower, but the surgical difficulty can be more challenging. For example, anatomical challenges, such as differences between the size of the implant and the alveolus postextraction, root length, height of root trunk, and divergence of roots make this surgical technique more difficult [10, 11].

To determine the possibility and prognosis of the implant placement in fresh extraction sockets prior to implant surgery, Smith and Tarnow [16] described a classification based on the morphology of the interradicular bone septa and its impact on the primary stability of the implant, permitting a more accurate presurgical planning. In 2016, Matsuda et al. [17] used a database of cone-beam imaging to evaluate the alveolar dimensions at molar sites and the possibility of immediate implant placement. The author reported that 46% of the sample (*n*=150) had 5 mm of engaging apical bone below the apex of the buccal mesial and distal roots that is compatible with an immediate implant procedure. Of the analyzed molars, 32% had a 2 mm distance from the sinus floor to the furcation and 5 mm between buccolingual roots, preventing an immediate implant approach. The rest of the molars were in an intermediate situation, with bone width greater than 5 mm between the roots but lacking height, having 2 mm to 4 mm from the root apex to the sinus floor, making an immediate implant approach technically more challenging. In the case presented in this report, the patient presented a type B socket [16] with a distance of 9 mm from the apex to the maxillary sinus and 8 mm between roots, presenting enough interradicular bone height and width to perform an immediate implant procedure.

In this clinical report, a guided bone regeneration technique was performed, which combined bovine xenograft grafting and PRF membrane placement on top of the extraction socket [18–20]. The available literature does not mention any potential benefit of this type of membrane on bone formation. Nevertheless, the authors used this approach to promote prolonged and continued release of growth factors and proteins by the extracellular matrix during the first few days, to strengthen the proliferation of blood vessels and accelerate the healing of soft tissue [21].

Even though there are a few, but similar, reported cases using an interradicular drilling technique before the extraction procedure, this clinical report contributes to the literature by increasing the scientific data regarding this technique and by adding a one-year clinical and radiographical follow-up after the placement of the prosthetics [8, 9]. Most of the articles found in this topic reported follow-ups only until the prosthetic delivery stage. This situation may be because the advantage of this technique is the surgical stage in which a primary stability and ideal three-dimensional positioning of the implants are attained. However, once osseointegration occurs, the behavior of peri-implant tissue should not differ from traditional procedures.

In Scarano's study [9], drilling in the interradicular bone septa before and after the extraction of molar roots was compared. The author concluded that using a guide on the position of the roots resulted in an ideal implant positioning (*p* < 0.05). Also, the primary stability of the implant based on a resonance frequency analysis had significantly higher implant stability quotient values (*p* < 0.05) as compared to the traditional technique of extraction, subsequent drilling, and immediate implant placement. However, we do not know whether other variables may affect these results. For example, the use of the ultrasound device is comparable to drilling with conventional rotary instruments, so these results cannot be generalized. Moreover, the criteria for inclusion were only molar sites with interradicular septa that had crown dimensions above 2.5 mm and apical dimensions above 3.5 mm, which does not necessarily represent what most prevails in the population nor mean that these minimum measurements will suffice to attain a primary stability.

The interradicular bone-drilling technique prior to dental extraction could be considered a simple yet useful modification to the standard drilling procedure. Its indications are absence of active infection, integrity of the roots, and sufficient remaining bone to allow an immediate implant approach [15]. Contraindications are dental mobility, due to severe loss of periodontal insertion, unfavorable root position, such as fused roots, ankyloses, and active infections [13, 15]. The authors have described that even active infections such as apical periodontitis do not lead to an increased risk of complications, as long as they are asymptomatic [4].

This procedure has an increased risk to alter the socket wall's morphology during the extraction procedure, leading to a deficient implant insertion. Therefore, careful extraction using desmotomes or ultrasonic appliances is advised to avoid any deformation of the interradicular bone that could lead to a modification of the bone-drilling path and alter the final implant position.

Researchers have even stated that this technique could be suitable to nonexpert clinicians, making it simpler to obtain a correct tridimensional position of the implant and primary insertion torque. This is also supported by studies that show the traditional approach where the level of expertise is key factor in the success of the procedure [12].

Some limitations of this technique are increased hardness of the root tissue, which may result in longer clinical time and greater risk of increasing intrabone temperature and of altering the normal healing because of the remains of dental tissue from drilling. Regarding the latter point, Davarpanah and Szmukler-Moncler [22] made a case report on 5 patients; according to the results, dental waste did not seem to interfere with implant osseointegration, but there was little scientific evidence on this latter point, so caution is recommended, with an emphasis on meticulous irrigation and surgical cleaning.

Although this technique is promising and the clinical yield has been good for the authors during intraoperation management and post-op check-ups, controlled randomized clinical testing is required, using a comparative method, to evaluate the benefits and limitations of this technique in the long term.
